# Baculovirus display of single chain antibody (scFv) using a novel signal peptide

**DOI:** 10.1186/1472-6750-10-80

**Published:** 2010-11-19

**Authors:** Kuntida Kitidee, Sawitree Nangola, Gaëlle Gonzalez, Pierre Boulanger, Chatchai Tayapiwatana, Saw-See Hong

**Affiliations:** 1 University Lyon 1, INRA UMR-754, Retrovirus & Comparative Pathology, 50, avenue Tony Garnier, 69366 Lyon Cedex 07, France; 2 Division of Clinical Immunology, Faculty of Associated Medical Sciences, Chiang Mai University, and Biomedical Technology Research Center, National Center for Genetic Engineering & Biotechnology, National Sciences and Technology Development Agency at the Faculty of Associated Medical Sciences, Chiang Mai University, Chiang Mai 50200, Thailand

## Abstract

**Background:**

Cells permissive to virus can become refractory to viral replication upon intracellular expression of single chain fragment variable (scFv) antibodies directed towards viral structural or regulatory proteins, or virus-coded enzymes. For example, an intrabody derived from MH-SVM33, a monoclonal antibody against a conserved C-terminal epitope of the HIV-1 matrix protein (MAp17), was found to exert an inhibitory effect on HIV-1 replication.

**Results:**

Two versions of MH-SVM33-derived scFv were constructed in recombinant baculoviruses (BVs) and expressed in BV-infected Sf9 cells, N-myristoylation-competent scFvG2/p17 and N-myristoylation-incompetent scFvE2/p17 protein, both carrying a C-terminal HA tag. ScFvG2/p17 expression resulted in an insoluble, membrane-associated protein, whereas scFvE2/p17 was recovered in both soluble and membrane-incorporated forms. When coexpressed with the HIV-1 Pr55Gag precursor, scFvG2/p17 and scFvE2/p17 did not show any detectable negative effect on virus-like particle (VLP) assembly and egress, and both failed to be encapsidated in VLP. However, soluble scFvE2/p17 isolated from Sf9 cell lysates was capable of binding to its specific antigen, in the form of a synthetic p17 peptide or as Gag polyprotein-embedded epitope. Significant amounts of scFvE2/p17 were released in the extracellular medium of BV-infected cells in high-molecular weight, pelletable form. This particulate form corresponded to BV particles displaying scFvE2/p17 molecules, inserted into the BV envelope via the scFv N-terminal region. The BV-displayed scFvE2/p17 molecules were found to be immunologically functional, as they reacted with the C-terminal epitope of MAp17. Fusion of the N-terminal 18 amino acid residues from the scFvE2/p17 sequence (N18E2) to another scFv recognizing CD147 (scFv-M6-1B9) conferred the property of BV-display to the resulting chimeric scFv-N18E2/M6.

**Conclusion:**

Expression of scFvE2/p17 in insect cells using a BV vector resulted in baculoviral progeny displaying scFvE2/p17. The function required for BV envelope incorporation was carried by the N-terminal octadecapeptide of scFvE2/p17, which acted as a signal peptide for BV display. Fusion of this peptide to the N-terminus of scFv molecules of interest could be applied as a general method for BV-display of scFv in a GP64- and VSV-G-independent manner.

## Background

The arsenal of HIV-1 antivirals available today includes a broad variety of drugs directed to viral targets which have a critical role at various steps of the virus life cycle. Inhibitors of virus-cell attachment and fusion, reverse transcription, protease-mediated maturation cleavage of viral protein precursors, and provirus integration into the host-cell genome, can be administered in multiple types of associations to minimize the emergence of resistance in highly active antiretroviral therapies (HAART). Among all the antiretroviral molecules, antibodies occupy a special position as they can inhibit HIV-1 replication by interfering with multiple steps of virus-cell interaction. Extracellular antibodies can neutralize HIV-1 at the early phase of cell attachment or entry of the virus [1]. On the other hand, intracellular antibodies (or intrabodies) can block virus replication by interfering with different processes, such as intracellular trafficking of incoming virions or assembly and egress of the virus progeny. The design of virus-resistant cells via intracellular expression of specific single chain fragment variable (scFv) antibodies directed to the virus has been successfully used to block HIV-1 replication *in vitro *[2-4]. The viral proteins which have been targeted by these intrabodies include structural proteins, such as the envelope glycoprotein gp120 [5] or the matrix protein MAp17 [6], the viral enzyme reverse transcriptase [7], and the auxiliary proteins Tat [8,9] and Vif [10,11].

The baculovirus (BV) *Autographa californica *multiple nucleopolyhedrovirus (AcMNPV) is an insect virus with a large double-stranded DNA genome packaged in a membrane-enveloped, rod-shaped protein capsid [12]. BVs have been extensively used over two decades as expression vectors for the production of recombinant proteins in insect cells [13]. The current interest of BVs resides in their promiscuous nature as gene transfer vectors, capable of transducing a large repertoire of established and primary cells, of both mammalian and nonmammalian origins [14,15]. Recombinant BVs carrying nonviral glycoproteins fused or nonfused to their own envelope glycoprotein GP64 have been advantageously used in the baculovirus-display technology and its multiple biological and therapeutic applications [16,17]. For example, fusion of scFv specific for the carcinoembryonic antigen (CEA) to GP64 conferred to the BV vector displaying scFv-CEA a targeting and binding specificity to CEA-expressing cells [18,19]. However, the fusion to GP64 restricts the display to the poles of the virions as well as the number of copies of fusion proteins, and other strategies using fusion to VSV-G glycoprotein have therefore been proposed [16,17,20,21].

It has been shown that the intracellular expression of a scFv derived from a monoclonal antibody (MH-SVM33) directed to a highly conserved C-terminal epitope of the HIV-1 MAp17 domain [22,23], scFv/p17, resulted in an efficient antiviral effect, as determined using CAp24-based ELISA and reverse transcription assays [6]. The MH-SVM33 epitope was localised near the MAp17-CAp24 junction and has been found to be accessible on recombinant Gag precursor (Pr55Gag), as shown by the high level of immunoreactivity of virus-like particles (VLP) produced in Sf9 cells and analyzed *in situ *by immuno-electron microscopy [24]. However, the exact molecular mechanism of the scFv/p17-mediated inhibitory activity has yet to be determined, since the MAp17 protein is involved in multiple viral functions within the infected cell (reviewed in [25]).

The original goal of our study was to try and elucidate the mechanism of the scFv/p17-mediated inhibitory activity, and its potential use for diagnostic or therapeutic applications. Insect cells infected by AcMNPV expressing Pr55Gag (AcMNPV-Pr55Gag) have been shown to produce vast amounts of VLP mimicking immature virions [26]. AcMNPV-Pr55Gag-infected Sf9 cells represent a convenient model for studying HIV-1 assembly [24,27-32], coencapsidation of Gag and partner proteins [33-35], and Gag processing by viral protease coexpressed in *trans *[36,37]. We constructed two recombinant BVs expressing scFv/p17 in two different formats. The N-myristoylation-competent version scFvG2/p17 carried a sequence which started with the N-terminal dipeptide Met-Gly, while the N-myristoylation-incompetent version scFvE2/p17 started with Met-Glu. Both scFV were incapable of blocking the assembly of VLP, or be copackaged with Pr55Gag into VLP. However, we found that scFvE2/p17 was incorporated into the baculoviral envelope (BV-E2/p17), and retained its anti-MAp17 functionality when displayed at the surface of BV particles. BV-E2/p17 represented therefore a potential biological tool for depletion of soluble MAp17 protein, or/and for competition with HIV-1 MAp17 receptors in *in vitro *or *ex vivo *experiments [25]. Interestingly, we found that another scFv molecule, referred to as scFv-M6-1B9 [38,39] and directed towards CD147 (also known as M6, OK, 5F7, TCSF, Basigin or EMMPRIN), was also displayed at the surface of baculoviral particles when fused to the same N-terminal octadecapeptide sequence from scFvE2/p17, abbreviated N18E2. This suggested that the N-terminal octadecapeptide sequence N18E2 carried the BV envelope addressing function and acted as a signal peptide for BV display. The fusion of this octadecapeptide sequence to the N-terminus of scFv molecules of biological interest could be used as a general strategy for BV-display, and as an alternative to fusion to GP64 or VSV-G.

## Methods

### Insect cells and baculovirus infection

*Spodoptera frugiperda *Sf9 cells were maintained as monolayers at 28°C in Grace's insect medium supplemented with 10% fetal bovine serum (FBS, Sigma), penicillin (200 U/ml), and streptomycin (200 μg/ml; Gibco-Invitrogen). They were infected with one, or coinfected with two or more recombinant BVs simultaneously at a multiplicity of infection (MOI) ranging from 2.5 to 20 pfu/cell, as previously described [24,28].

### Construction of scFv against HIV-1 MAp17 and CD147

The hybridoma cell line MH-SVM33C9/ATCC HB-8975 was obtained from the American Type Culture Collection (ATCC, Manassas, VA). The monoclonal antibody MH-SVM33 reacts with the conserved epitope ^121^DTGHSSQVSQNY^132 ^at the C-terminus of the matrix protein (MAp17) of HIV-1 [22,23]. Total RNA was extracted from hybridoma cells using the RNeasy Mini kit (Qiagen Inc., Hilden, Germany), and the first strand of cDNA was synthesized using the oligodT-18 primer of the Transcriptor High Fidelity cDNA synthesis kit (Roche, Mannheim, Germany). The variable regions (V) of heavy (V_H_) and light chains (V_L_) were then amplified from cDNA using specific forward (Fw) and reverse (Rev) primers. Fw-V_H_P17 (5'-ATATGCTAGCGGCCCAGGCGGCCCAGATCCAGTTGGTGCAGT-3') and Rev-V_H_P17 (5'-CGACCCTCCACCGCGGACCCGCCACCTCCAGACCCTCCGCCACCTGCA GAGACAGTGACCAGAGTCCC-3') were used for the V_H _fragment, and Fw-V_L_P17 (5'-GGGTCCGGCGGTGGAGGGTCGGATGTTGTGATGACCCAGACTCCA-3') and Rev-V_L_P17 (5'-ATATAAGCTTTCATTAAGCGTAGTCCGGAACGTCGTACGGGTACTGGCCGCCCTGGCCTT TGATTTCCAGC-3') for the V_L _fragment. The fragment encoding the single-chain antibody to MAp17 (scFv/p17) was constructed by overlapping PCR using Fw-V_H_P17 and Rev-V_L_P17 primers, both containing a *Sfi *I site (shown underlined). The construction and characterization of HA-tagged scFv M6-1B9 directed against CD147 have been described in a previous study [38,39].

### Recombinant BV

Foreign genes were inserted into the genome of AcMNPV, under the control of a chimeric AcMNPV-*Galleria mellonella *MNPV polyhedrin promoter in the case of recombinant HIV-1 Gag polyproteins [24,28,29,31,32], or under the control of the AcMNPV polyhedrin promoter in the case of pBlueBac4.5-derived vectors. AcMNPV-Pr55Gag, which expressed the N-myristoylated full-length Gag polyprotein (Pr55Gag) of HIV-1, has been described in detail in previous studies [30-32,36,40]. The recombinant AcMNPV expressing CAR (BV^CAR^), the high affinity receptor for Coxsackie B and Adenovirus, has been described and characterized in a previous study [41]. BV^CAR ^virions have been shown to display the CAR glycoprotein at their surface [41]. A DNA fragment coding for H_6_MA-CA, a non-N-myristoylated, carboxyterminal-truncated version of HIV-1 Gag polyprotein containing the matrix (MA) and capsid (CA) domains with an oligo-histidine (H_6_) tag at its N-terminus, was generating using the following pairs of primers: the first pair consisted of Fw primers 5'-CTAGCATGGGTGCGAGAG-3' and 5'-CATGGGTGCGAGAGCG-3' and the second pair consisted of Rev primers 5'-CTTACTACAAAACTCTTGCTTTATG-3' and 5'-GTACCTTACTAC AAAACTCTTGC-3'. Two PCR reactions were performed using the HIV-1 plasmid pNL4-3 as the template. The PCR products from both reactions were then mixed, denatured, and hybridized to obtain DNA fragments with *Nhe *I and *Kpn *I cohesive ends, competent for ligation to pBlueBac4.5-His intermediate vector linearized with *Nhe *I and *Kpn *I. Plasmid pBlueBac4.5-His was derived from pBlueBac4.5 (Invitrogen, San Diego, CA) by insertion of a sequence coding for the (H_6_) tag and a GSGSAS linker upstream to the *Nhe *I site. Sf9 cells were cotransfected with pBlueBac4.5-H_6_MA-CA and linearised BV DNA (Bac-N-Blue™ Transfection kit; Invitrogen). Positive Sf9 cells harboring recombinant BVs were isolated using the blue plaque selection method after beta-galactosidase staining. Recombinant BV, abbreviated BV-H_6_MA-CA, was isolated using the blue plaque selection method as above. Recombinant H_6_MA-CA protein was produced in Sf9 cells infected with BV-H_6_MA-CA. Sf9 cells were harvested at 48 h post infection (pi), and H_6_MA-CA protein recovered from clarified Sf9 cell lysate by affinity chromatography on Ni^2+^-NTA-agarose column (Qiagen, Hilden, Germany), as previously described [42]. Protein concentration in H_6_MA-CA samples was determined by Bradford protein assay (Pierce; Thermo Fisher Scientific Inc., Rockford, IL, USA). Recombinant H_6_MA-CA protein was used as the antigenic substrate for scFvG2/p17 and scFvE2/p17 in ELISA and co-immunoprecipitation experiments.

The DNA fragment encoding scFv/p17, obtained as described above, was cloned into the *Nhe *I and *Hind *III sites of the pBlueBac4.5 plasmid. The 5' and 3' ends of scFv/p17 fragment in the pBlueBac-scFv/p17 vector were then modified by oligonucleotide insertion at both *Nhe *I and *Hind *III sites, to obtain two versions of the scFv/p17 cDNA. One encoded the dipeptide Met-Gly at the N-terminus of scFv/p17, generating the scFvG2/p17 clone, the other the dipeptide Met-Glu, generating the scFvE2/p17 clone. At the 3' end of both clones, we inserted an oligonucleotide encoding the Influenza A virus hemagglutinin epitope YPYDVPDYA (HA tag). Sf9 cells were then cotransfected with the resulting plasmid harboring the scFv/p17 coding sequence and linearised BV DNA. Recombinant BVs were isolated using the blue plaque selection method, as described above

The DNA fragment for the HA-tagged scFv M6-1B9 was amplified using plasmid pComb3X-scFv-M6-1B9 as the template [39], with the M6-1B9 Fw primer (5'-GAGGAGGAGCTGGCCCAGGCGGCCCAGATCCAGTTGGTGCAGTCTGGAGAGCTAGTGATGACCCAGACTCCAGC-3') encoding the N-terminal 18 amino acids of scFvE2/p17 (^1^MEASLAAQAAQIQLVQSG^18^), and the M6-1B9 Rev primer (5'-CTCCTCCTCGGCCGCCCTGGCCACTAGTGACAGATGGGGCTG-3'). The scFv-M6-1B9 was then cloned into the *Sfi *I site of *Sfi *I-restricted pBlueBac-scFvE2. The recombinant BV was isolated as described above, and abbreviated BV-N18E2/M6.

### Isolation of BV particles

Concentrated stocks of recombinant BV expressing scFv/p17 molecules, BV-scFvG2/p17 and BV-scFvE2/p17, respectively, were prepared as follows [41]. Infected Sf9 cell culture supernatants were harvested at 50 to 60 h post infection (pi), and clarified by centrifugation at 2,400 rpm and 4°C for 10 min. Aliquots (11-ml) of clarified culture supernatant were subjected to ultracentrifugation at 28,000 rpm for 1 h at 4°C through a 1-ml sucrose cushion (20% sucrose, w:v in PBS) in Beckman SW41 rotor. Each baculoviral pellet was resuspended by gentle shaking in sterile phosphate-buffered saline (PBS) overnight at 4°C (100 μl per centrifuge tube). The titers of BV suspensions ranged usually between 5 × 10^9 ^and 1 × 10^10 ^pfu/ml, as determined by plaque titration in Sf9 cells (pfu/ml), which corresponded to 1 × 10^12 ^to 5 × 10^12 ^BV physical particles per ml [12,41]. When needed, e.g. for electron microscopy, BV particles were further purified by isopycnic ultracentrifugation in linear sucrose-D_2_O gradient [35,40]. Gradients (10-ml total volume, 30-50%, w:v) were generated from a 50% sucrose solution made in D_2_O buffered to pH 7.2 with NaOH, and a 30% sucrose solution made in 10 mM Tris-HCl, pH 7.2, 150 mM NaCl, 5.7 mM Na_2_EDTA. The gradients were centrifuged for 18 h at 28,000 rpm in a Beckman SW41 rotor. 0.5 ml-fractions were collected from the top, and proteins analyzed by SDS-PAGE and Western blotting with the required antibodies. BV particles were recovered in fractions with an apparent density ranging from 1.08 to 1.15.

### Cell fractionation and scFv purification

BV-infected Sf9 cells were harvested between 48 and 72 h pi. After cell lysis, cellular fractionation was performed using the FractionPREP™ Cell Fractionation System (Medical & Biological Laboratories Co. Ltd., Nagoya, Japan). Four subcellular protein fractions were thus obtained, cytosol, nucleus, membrane/particulate and cytoskeletal fractions. The scFvE2/p17 protein was purified by affinity selection on anti-HA tag antibody Affimatrix gel (Roche Applied Science, IN, USA), following the manufacturer's instructions. Each fraction eluted from the affinity gel, using elution buffer containing HA peptide (Roche Applied Science) as binding competitor to displace the bound proteins, was analyzed by SDS-PAGE, and scFvG2/p17 or scFvE2/p17 detected by Western blotting, using anti-HA tag antibody, as described below.

### Gel electrophoresis, membrane transfer and antibodies

Polyacrylamide gel electrophoresis of SDS-denatured protein samples (SDS-PAGE), and immunoblotting analysis have been described in detail in previous studies [34,35,40]. Briefly, proteins were electrophoresed in SDS-denaturing, 10%-polyacrylamide gel and electrically transferred to nitrocellulose membrane (Hybond™-C-extra; Amersham Biosciences). Blots were blocked in 5% skimmed milk in Tris-buffered saline (TBS) containing 0.05% Tween-20 (TBS-T), rinsed in TBS-T, then successively incubated with mouse monoclonal antibody to Influenza A virus hemagglutinin epitope YPYDVPDYA (HA tag antibody; Sigma, St Louis, MO, USA), or primary rabbit anti-Gag antibody, followed by relevant anti-IgG secondary antibodies, at working dilutions ranging from 1:1,000 to 1:10,000. Anti-HIV-1 Gag rabbit polyclonal antibody (laboratory-made; [35]) was raised in rabbit by injection of bacterially-expressed, GST-fused and affinity-purified carboxyterminally truncated Gag protein consisting of the full-length MA domain and the first seventy-eight residues of the CA domain (*Pst *I site; *gag*_Lai _sequence). Monoclonal antibody against baculoviral envelope GP64 glycoprotein, clone AcV1 was purchased from Santa Cruz Biotechnology (Santa Cruz Biotechnology, Inc., Santa Cruz, CA, USA). Apparent molecular weights were estimated by comparison with prestained protein markers (Precison Plus Protein™ Standards, Dual Color; BioRad Laboratories, Inc., Bio-Rad France). For protein quantification, blots were scanned and protein bands were quantitated by densitometry, using the VersaDoc image analyzer and the Quantity One program (BioRad).

### Indirect ELISA

The functionality of recombinant scFv/p17 was evaluated by their binding activity to the synthetic MAp17 peptide ^121^DTGHSSQVSQNY^132 ^(GenScript; Piscataway, USA) and H_6_MA-CA protein prepared as above, in standard indirect ELISA procedure. In brief, aliquots (100 μl) of MA p17 peptide solution at 50 μg/ml, or of H_6_MA-CA protein solution at 5 μg/ml in coating buffer (0.1 M NaHCO_3_, pH 9.6) were incubated overnight at 4°C in 96-well microtiter plates (NUNC, Roskilde, Denmark). The coated wells were blocked with 200 μl of blocking buffer (2% bovine serum albumin in TBS; TBS-BSA) for 1 h at room temperature (RT), then washed five times with washing buffer (0.05% Tween-20 in TBS; TBS-T). Aliquots (100 μl) from clarified cellular lysates of BV-G2/p17- or BV-E2/p17-infected Sf9 cells in blocking buffer was added to each well and incubation proceeded for 1 hr at RT. After five cycles of washing with TBS-T, monoclonal anti-HA tag antibody (Sigma) was added at dilution 1:2,500 in TBS-BSA, and incubated for 1 hr at RT. After extensive washing with TBS-T, the reaction was developed by addition of 100 μl p-nitrophenylphosphate substrate (Sigma), and OD measured at 405 nm using an ELISA plate reader at the optimum time. The antigen-binding activity of scFv-M6-1B9 and chimeric scFvE2/M6-1B9 was assessed in ELISA using immobilized recombinant CD147-biotin carboxyl carrier protein (BCCP) fusion protein as the antigen, produced as previously described [39].

### Competition ELISA

Microtiter plates (NUNC) were coated with 50 μl of 10 μg/ml of avidin in coating buffer (0.1 M NaHCO3, pH 6.8) and incubated overnight at 4°C in moist chamber. The coated wells were then blocked with 200 μl of blocking buffer (2% BSA in TBS) for 1 h at RT, then washed four times with washing buffer (0.05% Tween-20 in TBS). 50 μl-aliquots of 50 μg/ml of biotinylated-peptide 17.1 (Bp17.1) in blocking buffer were added to each well and incubated for 1 h at RT. In parallel, scFvE2/p17 purified by anti-HA tag affinity as described above was mixed with p17 peptide at the final peptide concentration of 1 μg/ml, and incubated for 1 h at RT. After washing the wells, the mixture was added to the wells and incubated for 1 h at RT. Bound scFvE2/p17 was monitored by adding 50 μl of HRP-conjugated monoclonal anti-HA tag antibody (Sigma) at dilution 1:1,000 in blocking buffer. The wells were washed four times prior to the addition of 50 μl of 3,3',5,5'-tetramethyl-benzidine (TMB) substrate. Reaction was stopped by addition of 50 μl of 1 N HCl, and optical densities (OD) at 450 nm were measured using an ELISA plate reader. The percentage of inhibition (PI) was given by the following formula: PI = 100-[(B:Bo) × 100], where B and Bo were the OD values for scFvE2/p17 with and without inhibitor, respectively.

### Co-immunoprecipitation

Sf9 cells co-infected with BV-E2/p17 (or BV-G2/p17) and BV-H_6_MA-CA were harvested by centrifugation at 5,000 rpm for 10 min at 4°C. The cell pellets were washed 3 times with cold TBS, then resuspended in lysis buffer (1% Brij-35 in TBS) and incubated on ice for 45 min. The cell lysates were clarified by centrifugation at 13,000 rpm for 1 h at 4°C, and supernatants added to pre-washed anti-HA tag affinity gel. After incubation overnight at 4°C with gentle shaking, the resin was washed 3 times with washing buffer (20 mM Tris, pH7.5, 0.1 M NaCl, 0.1 mM EDTA, 0.05% Tween20). Immunocaptured proteins were eluted from the affinity gel by heating in SDS-containing buffer (20 mM Tris pH 7,5, 2 mM EDTA, 5% SDS, 20% glycerol, 200 mM DTT, 0.02% bromophenol blue) at 100°C for 5 min. The resin was pelleted by centrifugation and the supernatant analyzed by SDS-PAGE and Western blotting, using anti-His_6 _tag and anti-HA tag primary antibodies, both purchased from Sigma.

### Immunofluorescence (IF) microscopy

BV-infected Sf9 cell monolayers were harvested at 48 h pi, fixed with 3% paraformaldehyde in phosphate buffered saline (PBS) and permeabilized in 0.2% (v/v) Triton X-100 in PBS. Cells were blocked with 3% BSA in PBS (PBS-BSA), and HA-tagged scFv/p17 detected by reaction with mAb anti-HA (1:10,000 in PBS-BSA) and Alexa Fluor^® ^488-labeled goat anti-mouse IgG antibody (Molecular Probes, Invitrogen). For double labeling of scFv/p17 and Gag proteins, cell samples were reacted with rabbit anti-Gag antibody (1:1,000 in PBS-BSA) and Alexa Fluor^® ^546-labeled goat anti-rabbit IgG (Molecular Probes, Invitrogen). Samples were treated with DAPI and mounted on slides. For conventional IF microscopy, images were acquired using an Axiovert 135 inverted microscope (Zeiss) equiped with an AxioCam video camera. For confocal microscopy, samples were analyzed using a Leica TCS SP2 confocal microscope.

### Flow cytometry

ScFv-expressing Sf9 cells were resuspended in 200 μl PBS and incubated with monoclonal anti-HA tag antibody for 1 h at room temperature (RT), and at the dilution recommended by the manufacturer (Sigma), then pelleted. The cell pellet was resuspended in 200 μl PBS and reacted with Alexa Fluor^® ^488-labeled goat anti-mouse IgG antibody (Molecular Probes, Invitrogen). The cell suspension was then diluted with 10 volumes of PBS, and analyzed by flow cytometry using a BD FACSCanto™ II cytometer (Becton Dickinson Biosciences). At least 10,000 events were acquired for each experiment using the DIVA 6 software (Becton Dickinson).

### Electron microscopy (EM)

Specimens were processed for EM and observed as previously described [41]. In brief, pelleted virions of BV-E2/p17 and BV-G2/p17 were resuspended in 20 μl-aliquots of 0.14 M NaCl, 0.05 M Tris-HCl buffer, pH 8.2, and adsorbed onto carbon-coated formvar membrane on nickel grids. The grids were incubated with primary antibody (anti-HA tag monoclonal antibody) at a dilution of 1: 50 in TBS for 1 h at room temperature (RT). After rinsing with TBS, the grids were post-incubated with 20-nm colloidal gold-tagged goat anti-mouse IgG antibody (British Biocell International Ltd, Cardiff, UK; diluted to 1: 50 in TBS) for 30 min at RT. After rinsing with TBS, the specimens were negatively stained with 1% uranyl acetate in H_2_0 for 1 min at RT, rinsed again with TBS, and examined under a JEM 1400 Jeol electron microscope equiped with an Orius-Gatan digitalized camera (Gatan France, 78113 Grandchamp).

### Statistics

Results were expressed as mean ± SEM. of *n *observations. Sets of data were compared with an analysis of variance (ANOVA) or a Student's *t *test. Differences were considered statistically significant when P < 0.05. Symbols used in figures were (*) for P < 0.05, (**) for P < 0.01, (***) for P < 0.001, and ns for no significant difference, respectively. All statistical tests were performed using GraphPad Prism version 4.0 for Windows (Graphpad Software).

## Results

### Expression and characterization of scFvG2/p17 and scFvE2/p17 molecules in recombinant BV-infected Sf9 cells

The monoclonal antibody secreted by the MH-SVM33C9 hybridoma cell line reacts with the highly conserved and accessible epitope ^121^DTGHSSQVSQNY^132 ^corresponding to the C-terminus of HIV-1 MAp17 [22-24]. A single chain antibody derived from MH-SVM33 (scFv/p17) and expressed intracellularly showed an inhibitory effect on HIV-1 replication and virus release [6]. We generated two scFv/p17 subclones from the MH-SVM33C9 hybridoma cell line, scFvE2/p17 and scFvG2/p17, of which the full sequence could be communicated upon request. The N-terminal octadecapeptide sequence in scFvE2/p17 read ^1^MEASLAAQAAQIQLVQSG^18 ^, and ^1^MGLAAQAAQIQLVQSGPE^18 ^in scFvG2/p17. Both subclones were also modified at the C-terminus by the addition of a HA-tag, the Influenza A virus hemagglutinin epitope YPYDVPDYA, and when inserted into the baculoviral genome, generated two recombinant BVs, BV-E2/p17 and BV-G2/p17, respectively. The rationale for the N-terminal modification was that the dipeptide Met-Gly at the N-terminus of scFvG2/p17 represented a N-myristoylation signal which would promote the addressing scFvG2/p17 to the same compartment as the N-myristoylated MAp17 protein and Pr55Gag polyprotein. We have shown in previous studies that the Met-Gly signal was functional in insect cells in terms of recognition by N-myristoyl transferase, and that N-myristoylated glycine residue at the N-terminus of Pr55Gag is a prerequisite for plasma membrane addressing of unprocessed Pr55Gag, and budding and egress of membrane-enveloped VLP from Sf9 cells [24,27-36,40].

**Figure 1:**
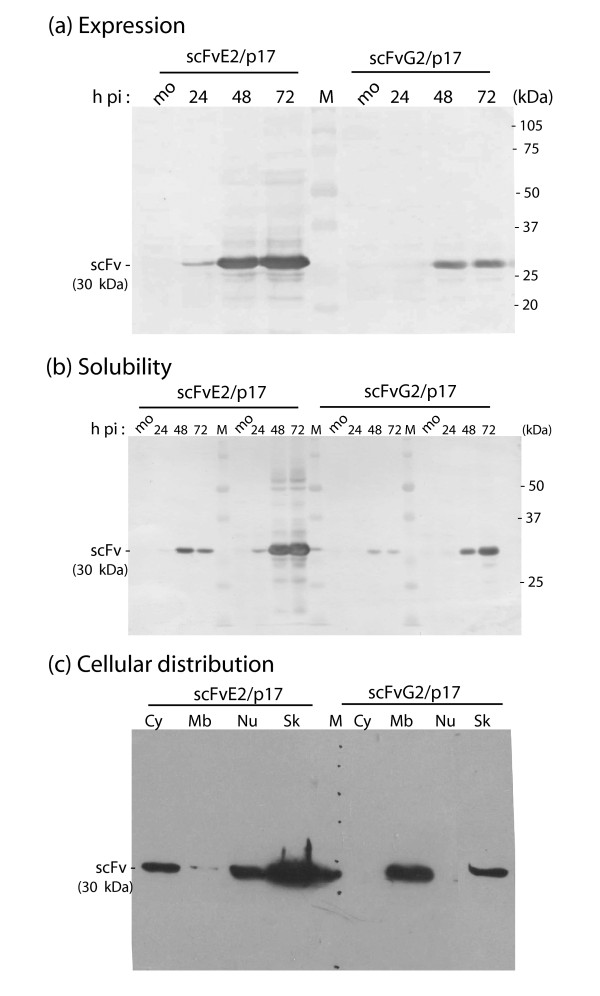
**Expression, solubility and cellular localization of scFvE2/p17 and scFvG2/p17 in Sf9 cells**. **(a)**, ***Level of expression***. Sf9 cells were mock-infected (lanes mo), or infected with BV-E2/p17 or BV-G2/p17, and harvested at 24, 48 and 72 h pi, as indicated on top of the panel. Whole cell lysates were analyzed by SDS-PAGE and Western blotting using anti-HA tag antibody. **(b), *Solubility***. Lysates of mock-infected cells (mo) or BV-E2/p17- or BV-G2/p17-infected cells harvested at 24, 48 and 72 h pi, as indicated on top of the panel were clarified by centrifugation, and soluble fraction (S) and pelletable material (P) analyzed as above. M, molecular mass markers, with their apparent molecular masses indicated in kilodaltons (kDa) on the right side of the blots. **(c), *Cell fractionation***. Sf9 cells infected with BV-E2/p17 or BV-G2/p17 as indicated on top of the panel were harvested at 48 h pi and processed for cell fractionation into cytosolic compartment (Cy), membranes (Mb), nuclear compartment (Nu) and cytoskeletal-associated proteins (Sk). Subcellular fractions were analyzed by SDS-PAGE and Western blotting using anti-HA tag antibody.

Sf9 cells infected with BV-G2/p17 or BV-E2/p17 were harvested at 24, 48 and 72 h post infection (pi), lysed in hypotonic buffer, and the kinetics of synthesis of scFvG2/p17 and scFvE2/p17 was analyzed by SDS-PAGE and Western blotting using anti-HA tag antibody. The extracellular culture medium was analyzed in parallel. We found that scFvE2/p17 protein (migrating with an apparent molecular mass of 30 kDa) was detectable as early as 24 h, and was maximal at 72 h pi. In contrast, scFvG2/p17 was detected at later times (48 h pi), and in 4- to 5-fold lower amounts, compared to scFvE2/p17 at 48-72 h pi (Figure 1a). However, both scFvE2/p17 and scFvG2/p17 proteins were stable over a period of 72 h, with no major breakdown products detected in the Western blot patterns.

Cell lysates were then clarified by centrifugation, and scFvG2/p17 and scFvE2/p17 were probed in soluble fraction (S) and insoluble pellet (P), respectively. We found that 15-20% of the whole recombinant scFvE2/p17 protein synthesized was recovered as soluble material, as determined by scanning and densitometric analysis of the protein bands on blots, with a maximum at 48 h pi (Figure 1b; leftmost half of the panel). The proportion of soluble scFvG2/p17 was lower (Figure 1b; rightmost half), with only 8-10% of scFvG2/p17 protein recovered in the soluble fraction. The difference in the intracellular levels of the two recombinant proteins could not be explained by a higher level of scFvG2/p17 secretion, compared to scFvE2/p17, as no scFvG2/p17 protein was detected in the culture medium at any time pi. Only scFvE2/p17 protein was detectable in the extracellular medium, as described below.

### Cellular distribution of scFvG2/p17 and scFvE2/p17 in recombinant BV-infected Sf9 cells

We next performed cell fractionation to determine in which subcellular compartments the majority of scFvE2/p17 and scFvG2/p17 proteins were localized. Of note, the distinction between cytosol (Cy), membranes and organelles (Mb), nucleus (Nu) and cytoskeleton (Sk) was only operational, and did not preclude probable cross contaminations between different fractions. With this restriction in mind, scFvE2/p17 was found to be associated with the cytosolic fraction and nuclear pellet in similar amounts (ca. 20-25% each), but larger quantities (50-60%) were recovered in the insoluble fraction of cytoskeletal proteins (Figure 1c; leftmost half of the panel). Only small amounts of scFvE2/p17 were detected in the membrane fraction (Figure 1c, Mb lane). The pattern of subcellular localization was different for the scFvG2/p17 protein, which was undetectable in the cytosolic and nuclear fractions, but distributed unequally between membrane and cytoskeletal fractions, two-thirds and one-third of the total, respectively (Figure 1c, rightmost half of the panel). Thus, the N-terminal G^2 ^mutation conferred two new properties to scFvG2/p17, compared to its scFvG2/p17 counterpart, (i) a lower level of expression, and (ii) a relocation to and strong association with the membranal fraction. The membrane association of scFvG2/p17 determined by cell fractionation was *a priori *consistent with the membrane targeting expected for a N-myristoylated protein.

**Figure 2:**
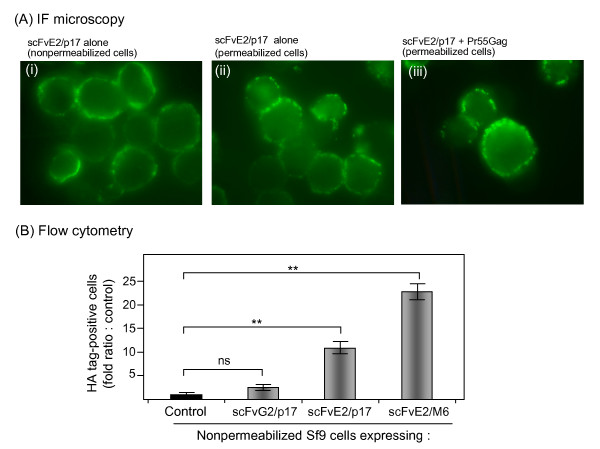
***In situ *analysis of scFvE2/p17 and scFvG2/p17 proteins in Sf9 cells**. **(A), *Immunofluorescence (IF) microscopy***. Sf9 cells expressing scFvE2/p17 alone (**i, ii**), or coexpressing scFvE2/p17 and Pr55Gag (**iii**), were harvested at 48 h pi and nonpermeabilized **(i)**, or permeabilized with Triton X-100 **(ii, iii)**. Cells were reacted with anti-HA tag monoclonal antibody followed by Alexa Fluor^® ^488-labeled complementary antibody. **(B), *Flow cytometry***. Nonpermeabilized Sf9 cells expressing scFvE2/p17, scFvG2/p17 or the scFv-N18E2/M6 chimera were harvested at 48 h pi, reacted with antibodies as in (A), and analyzed by flow cytometry. Results shown were the proportion of HA tag-positive cells, expressed as the fold ratio over the values of control cells, attributed the value of 1. Control consisted of BV^CAR^-infected cells, i.e. cells expressing irrelevant membrane glycoprotein. BV^CAR ^was a recombinant BV expressing the human CAR glycoprotein, and BV^CAR^-infected cells released CAR-displaying virions in the extracellular medium [41]. Average of three separate experiments, m ± SEM; (**), *P *< 0.01; ns, not significant.

Cellular localization of scFvG2/p17 and scFvE2/p17 was then studied *in situ*. BV-infected Sf9 cells were harvested at 48 h pi and examined in immunofluorescence (IF) microscopy or analyzed by flow cytometry using anti-HA tag antibody, with or without membrane permeabilization with detergent. Fluorescent signal of scFvE2/p17 was detected in both nonpermeabilized (Figure 2A, i) and Triton X100-permeabilized cells (Figure 2A, ii), whereas scFvG2/p17 fluorescence was only detectable in permeabilized cells (not shown). Flow cytometry of HA tag-positive cells confirmed the accessibility of scFvE2/p17 at the surface of nonpermeabilized cells, and the absence of significant amounts of scFvG2/p17 molecules at the cell surface (Figure 2B). Considering the poor recovery of scFvE2/p17 in the membranal fraction upon cell fractionation (refer to Figure 1c), the results of IF microcopy and flow cytometry suggested that the majority of scFvE2/p17 molecules were addressed to the plasma membrane and highly accessible at the cell surface, but proned to dissociate from the membrane upon cell disruption and fractionation, and to relocate into the soluble fraction. In contrast, we observed a major intracellular retention of most of the scFvG2/p17 molecules. Together with the data of cell fractionation (Figure 1c), this suggested that scFvG2/p17 protein was in majority sequestered in the intracellular membrane network.

When Sf9 cells were coinfected with two recombinant BVs to coexpress scFvE2/p17 and N-myristoylated Pr55Gag, we detected no significant change in the IF pattern of scFvE2/p17, compared to single infected cells expressing scFvE2/p17 alone (Figure 2A, compare panels ii and iii). This suggested that there was no major cellular redistribution of the scFvE2/p17 molecules in the presence of the Gag precursor. Similarly, no cellular redistribution of scFvG2/p17 was observed when coexpressed with Pr55Gag (not shown).

### Immunological characterization of extracellular scFvE2/p17 molecules: functionality and specificity

#### (i) Antigen recognition by scFvE2/p17 in vitro

**Figure 3:**
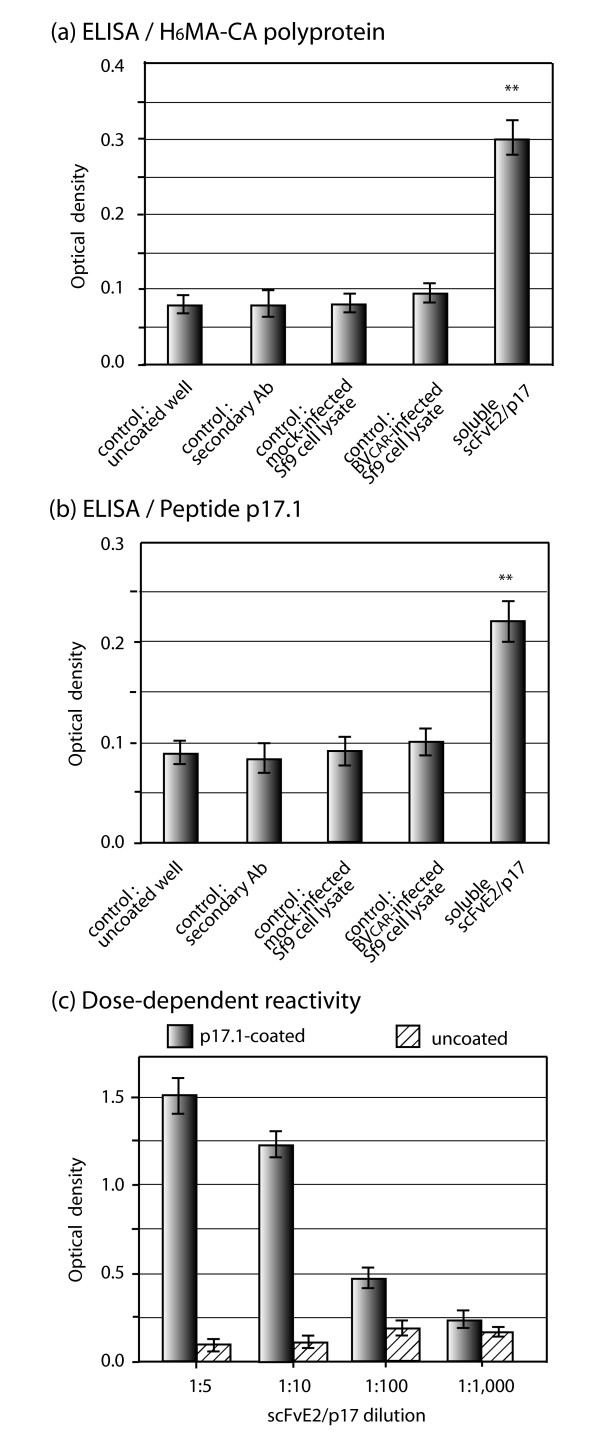
**Functionality of soluble scFvE2/p17: antigen recognition**. Sf9 cells were mock-infected or infected with BV^CAR ^(expressing irrelevant recombinant protein), or BV-E2/p17, and harvested at 48 h pi. Sf9 cell lysates were clarified by centrifugation, and reacted in ELISA with immobilized antigen. **(a)**, H_6_MA-CA protein; **(b, c)**, synthetic peptide p17.1 (^121^DTGHSSQVSQNY^132 ^epitope). Negative controls were, from left to right: uncoated well, antigen-coated well without addition of scFvE2/p17-containing lysate, mock-infected cell lysate, and BV^CAR^-infected cell lysate, respectively. **(c)**, Dose-dependent immunoreactivity of scFvE2/p17 towards p17.1 peptide. Soluble scFvE2/p17 from Sf9 cell lysates was affinity-purified on anti-HA tag antibody-coupled agarose beads. Average of three separate experiments, m ± SEM; (**), *P *< 0.01; ns, not significant.

**Figure 4:**
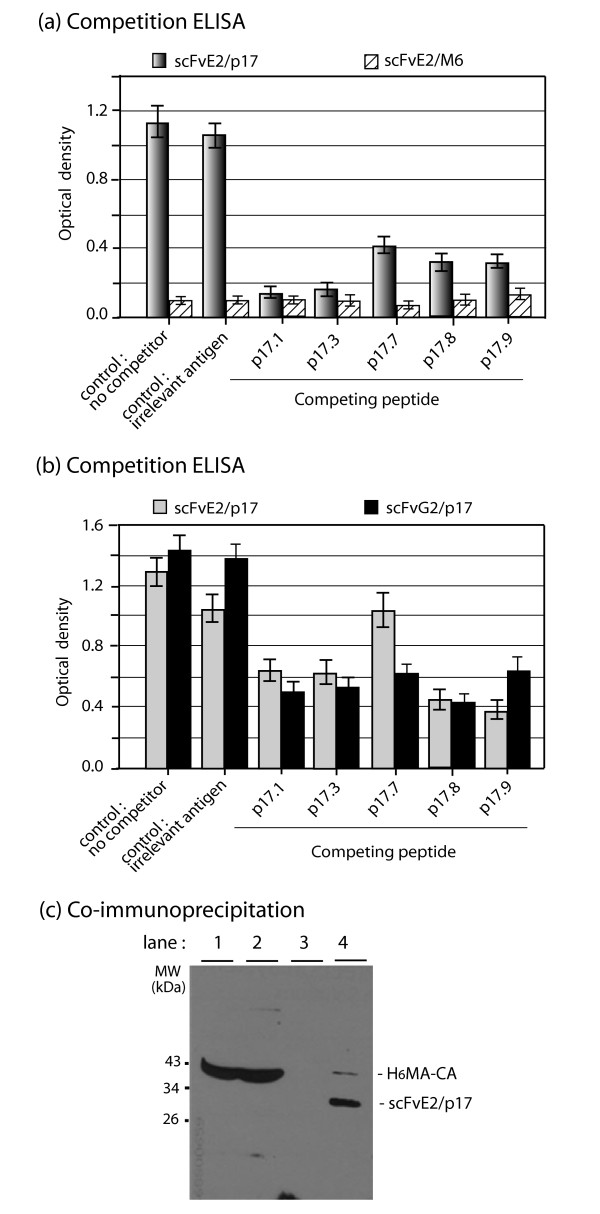
**Immunological characterization of scFvE2/p17: epitope specificity and affinity**. **(a)**, Soluble scFvE2/p17 from Sf9 cell lysates was analyzed in competition ELISA using solid support-adsorbed p17.1 versus soluble p17.1 (homologous, positive control), or versus MAp17 epitope variants p17.3, p17.7 and p17.9. Negative controls consisted of ELISA in the absence of competing peptide (no competitor), or ELISA in the presence of irrelevant antigen (CD147), or irrelevant scFv (scFv-M6-1B9). Average of three separate experiments, m ± SEM. **(b)**, Isolation of H_6_MA-CA-scFvE2/p17 complexes. Lysates of cells coexpressing H_6_MA-CA and HA-tagged scFvE2/p17 were separated on affinity matrix consisting of anti-HA tag antibody-coupled agarose beads, and fractions analyzed by SDS-PAGE and Western blotting, using anti- His_6 _tag and anti-HA tag antibodies. Lane 1, whole cell lysate; lane 2, flow-through fraction; lane 3, column wash; lane 4, fraction eluted with SDS-PAGE loading buffer.

Since a significant proportion of scFvE2/p17 molecules occurred as soluble intrabody in the cytosolic fraction, we tested lysates of BV-E2/p17-infected Sf9 cells for the antigen binding activity of scFvE2/p17 in ELISA. Two types of immobilized antigens were used: a synthetic peptide p17.1, which reproduced the epitope sequence from HIV-1_LAI _MAp17 (^121^DTGHSSQVSQNY^132^), and corresponded to the immunogen used to generate MH-SVM33; a recombinant H_6_MA-CA protein of 39 kDa, which carried the same epitope as p17.9 (Table 1), but was embedded in the Gag polyprotein. The binding data from ELISA clearly showed that recombinant scFvE2/p17 reacted with its specific epitope in both configurations (Figure 3a, b), and in a dose-dependent manner (Figure 3c). Competition ELISA indicated that scFvE2/p17 bound to its epitope with a higher affinity when it was used as free p17.1 peptide, compared to the H_6_MA-CA polyprotein used at equivalent epitope molarities (Figure 4a), suggesting that scFvE2/p17 preferentially bound to cleaved matrix protein MAp17, compared to non processed Gag precursor.

**Table 1:** Affinity of scFvE2/p17 for different variants of the conserved C-terminal epitope of HIV-1 MAp17 (a).

Competitor	Epitope	Binding competition vs p17.1 (%)	Origin or HIV-1 isolate
Unrelated antigen (negative control)	CD147 (M6)	5.2 ± 2.9	-
17.1 (positive control)	DTGHSSQVSQNY	89.5 ± 1.6	LAI ^(b)^
17.3	DTGHSSQ**I**SQNY	74.7 ± 10.2	1M-1005 ^(c)^
17.7	DTGHSSQ**A**SQNY	44.0 ± 18.0	g22s2 ^(d)^
17.8	DTGHS**K**QVSQNY	81.5 ± 9.5	4 ^(e)^
17.9	DTG**NN**SQVSQNY	68.8 ± 2.2	pNL4.3 ^(f)^
Inverted p17.1	YNQSVQSSHGTD	3.8 ± 1.5	-

^(a) ^Competition for scFvE2/p17 binding between the dodecapeptide p17.1 (corresponding to residues 121-132 in the MAp17 from HIV-1_LAI_, used as immunogen) and other epitope variants was determined by ELISA, as examplified in Figure 4c. Data presented are m ± SEM (*n *= 3). Residues differing from the LAI prototype sequence are in bold.

^(b) ^[63].

^(c) ^[64].

^(d) ^[43].

^(e) ^[65].

^(f) ^[66].

#### (ii) HIV-1 strain-specificity of antigen recognition by scFvE2/p17 and scFvG2/p17

Although the MAp17 epitope recognized by the monoclonal antibody MH-SVM33 is highly conserved among HIV-1 strains, there are some subtle differences in amino acid sequence. To assess the stringency of scFvE2/p17 towards HIV-1 MAp17 variants, we tested five different MAp17 peptides versus the original peptide epitope p17.1 in competition ELISA (Table 1). All MAp17 peptide variants competed with p17.1 to significant levels, considering that control homologous competition (bound p17.1 vs free p17.1) showed a 90% binding inhibition (Figure 4a, and Table 1). The lowest competition was observed for peptide p17.7, which corresponded to the MAp17 epitope of isolate g22s2, a molecular clone of HIV-1 isolated from HAART treated AIDS patients [43]. The difference between p17.7 and p17.1 corresponded to an Ala-Val mutation at position 128 in the MA domain. Valine carries a bulkier and more sterically hindered side chain, compared to alanine, and many examples have been reported in the literature of highly deleterious effects provoked by Ala-to-Val substitutions, in terms of protein conformation and function [44,45]. Competition ELISA was also performed using p17.1 versus a synthetic peptide of identical composition but of inverted sequence, YNQSVQSSHGTD. No competition was observed (Table 1), implying that N-to-C orientation of the peptide sequence and the C-terminal position of tyrosine residue-132 were crucial for the recognition of the epitope by scFvE2/p17.

The affinity and binding specificity of scFvG2/p17 to the different MAp17 peptides was also tested using competition ELISA, as above. Since only a minor fraction of recombinant scFvG2/p17 protein was recovered in the soluble cytosolic fraction, clarified lysates of cells expressing scFvG2/p17 were concentrated by adsorption onto anti-HA tag-agarose gel, and specific elution of scFvG2/p17 protein was carried out using HA peptide-containing buffer. No significant difference was observed between scFvG2/p17 and scFvE2/p17 in terms of reactivity with MAp17 epitope variants (Figure 4b). The lack of difference in the antigen-binding function of soluble scFvG2/p17 and scFvE2/p17 indicated that the apparent poorer solubility of scFvG2/p17 compared to scFvE2/p17 was likely due to its addressing to membranal and cytoskeletal compartments, and not to major changes in its overall conformational structure and/or complementary-determining regions. This underlined the importance of the N-terminal sequence, which harboured the only difference between scFvG2/p17 and scFvE2/p17.

### Functionality of scFvE2/p17 and scFvG2/p17 as intrabodies

#### (i) Antigen binding

Although our recombinant scFvE2/p17 was active *in vitro *in ELISA, we sought to determine whether intracellular scFvE2/p17 molecules could bind *in situ *to their MAp17 epitope embedded in Gag polyprotein. Sf9 cells were co-infected with two recombinant BVs, one expressing scFvE2/p17 (or scFvG2/p17), the other expressing the non-N-myristoylated Gag polyprotein substrate H_6_MA-CA. Possible antigen-intrabody complexes present in cell lysates were isolated by affinity chromatography on anti-HA tag-agarose gel. The pattern of SDS-PAGE and Western blot analysis showed that most of the Gag polyprotein H_6_MA-CA was recovered in the flow-through fraction, and that only a minor fraction bound to the column as H_6_MA-CA-scFvE2/p17 complex (Figure 4b, lane 4). This suggested that scFvE2/p17 intrabody and the non-N-myristoylated H_6_MA-CA protein segregated in separate cellular compartments and that only a small proportion of the scFvE2/p17 molecules could bind to H_6_MA-CA. This might also suggest that scFvE2/p17 intrabody had a relatively low affinity for its Gag-embedded p17 epitope, compared to fully processed protein MAp17 with accessible p17 epitope at its C-terminus, or free C-terminal p17 peptide. These different hypotheses are not mutually exclusively. No detectable antigen-scFv complex was found with scFvG2/p17 (not shown), which confirmed that the majority of scFvG2/p17 accumulated in an insoluble form, and/or in a cellular compartment inaccessible to cytoplasmic Gag polyprotein.

#### (ii) Effect of scFvE2/p17 on VLP production

As shown earlier by IF microscopy, Pr55Gag had no significant influence on the cellular distribution of scFvE2/p17 protein (refer to Figure 2A, iii). To further study the mechanism of HIV-1 antiviral effect of MH-SVM33-derived scFv/p17 [6], we investigated the possibility that scFvE2/p17, via its binding to MAp17, could modify the intracellular trafficking and assembly pathway of Pr55Gag molecules. Sf9 were co-infected with two recombinant BVs, one expressing the N-myristoylated Pr55Gag precursor, the other scFvE2/p17. Pr55Gag synthesis was analyzed in whole cell lysates, and VLP production assayed in parallel in the culture medium, as described in previous studies [34,35,40]. No negative effect on Pr55Gag protein synthesis and on VLP assembly was detected in the presence of scFvE2/p17 (data not shown). Possible coencapsidation of Pr55Gag and scFvE2/p17 molecules into VLP during the assembly process was also investigated. Extracellular VLP were isolated from the culture medium, and their protein composition analyzed by SDS-PAGE and Western blotting using anti-Gag and anti-HA tag antibodies: no scFvE2/p17 was detected in the VLP fraction (not shown). This was in contrast to the BV particle fraction, as shown below.

### Biophysical status of extracellular scFvE2/p17 protein: soluble versus particulate

**Figure 5:**
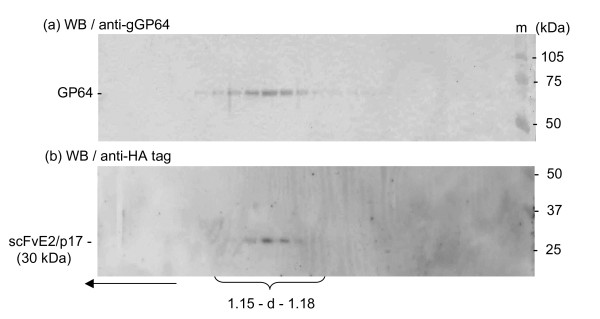
**BV display of scFvE2/p17 (BV-E2/p17)**. Samples of culture medium of Sf9 cells infected with BV-E2/p17 were harvested at 72 h pi and analyzed by isopycnic ultracentrifugation in sucrose-D_2_0 gradients, as described under Materials and Methods. Gradient fractions were analyzed by SDS-PAGE and Western blotting, using **(a) **monoclonal antibody to the baculoviral GP64 envelope glycoprotein for detection of baculovirus progeny, and **(b) **anti-HA tag monoclonal antibody for detection of scFvE2/p17.

As mentioned above, a significant amount of scFvE2/p17 protein was recovered in the extracellular medium of BV-E2/p17-infected Sf9 cells. These extracellular scFvE2/p17 molecules might occur as soluble protein, or as particle-associated material, e.g. released within membrane microvesicles or exosomes, or associated with cell debris. In order to determine the status of extracellular scFvE2/p17 protein, samples from culture mediun were subjected to isopycnic ultracentrifugation analysis in sucrose-D_2_O density gradients [35,40]. Gradient fractions were analyzed by SDS-PAGE and Western blotting, using anti-baculoviral envelope glycoprotein GP64 and anti-HA tag antibodies. We found that the fractions positive for the C-terminal HA tag of scFvE2/p17 coincided with the anti-GP64-reacting fractions, and corresponded to particulate material sedimenting with the apparent density of BV particles of the viral progeny, d = 1.15-1.08 (Figure 5). This suggested that scFvE2/p17 was associated with the BV particles, either coencapsidated with the baculoviral proteins or inserted into the viral envelope. In the latter case, scFvE2/p17 molecules could be exposed at the surface of the BV particles with their active site accessible for epitope binding.

### Immuno-EM analysis of baculoviral progeny of BV-E2/p17

**Figure 6:**
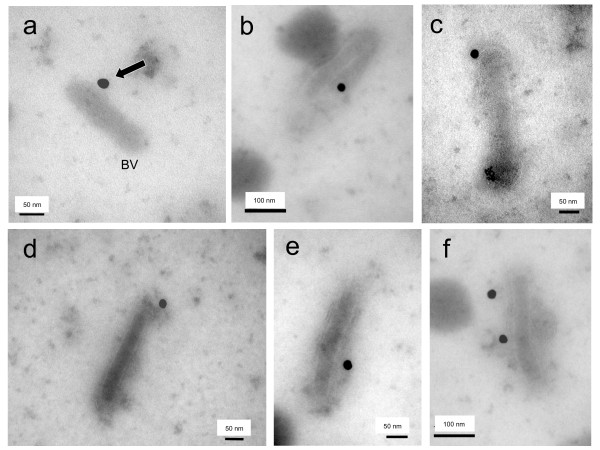
**Immuno-electron microscopy of BV particles carrying scFvE2/p17 (BV-E2/p17)**. Samples of baculovirus progeny recovered from the culture medium of Sf9 cells infected with BV-E2/p17 and harvested at 72 h pi were deposited on grids and negatively stained with uranyl acetate. Specimens were reacted with anti-HA tag monoclonal antibody, followed by anti-mouse IgG antibody coupled to 20-nm colloidal gold grains. Different fields are presented in panels (a) to (f). Arrow in **(a) **points to a gold grain associated with a BV particle (BV). In **(f)**, two gold grains are seen associated with one BV particle.

In order to confirm the reality of this surface exposure, samples of extracellular medium of BV-G2/p17- and BV-E2/p17-infected Sf9 cells were analyzed by isopycnic ultracentrifugation in sucrose-D_2_O gradients, and fractions sedimenting at 1.15-1.08 were deposited on grids, immunogold labeled with anti-HA tag antibody, and observed under the electron microscope. BV-G2/p17 virions were never seen associated with gold grains, and most immunogold labeling was found at distance from BV particles, and corresponded to nonspecific, background labeling (not shown). The absence of anti-HA labeling of BV-G2/p17 virions in immuno-EM was consistent with the absence of detectable scFvG2/p17 protein in the extracellular medium, as mentioned above. Under the same experimental conditions however, anti-HA tag immunogold labeling was found to be associated with virions of BV-E2/p17 (Figure 6). The immuno-EM analysis therefore confirmed that scFvE2/p17 molecules were truely incorporated into the baculoviral envelope. Such incorporation of foreign proteins into the baculoviral envelope has already been described with human membrane glycoprotein CAR [41].

### Immunological functionality and topology of scFvE2/p17 displayed on the baculoviral envelope

**Figure 7:**
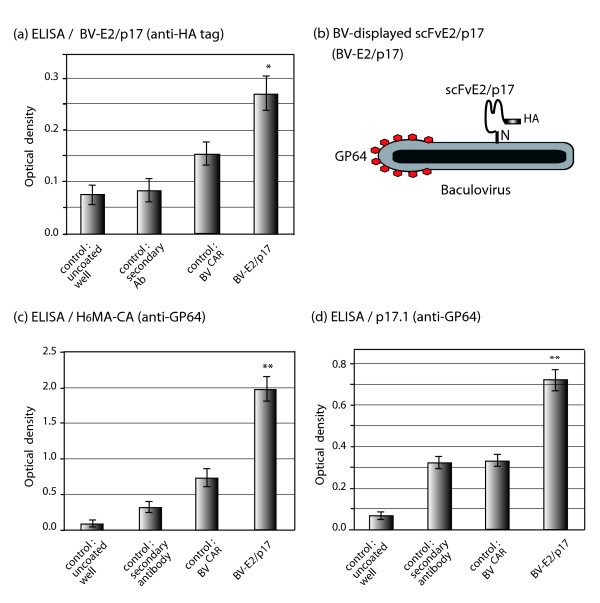
**Topology and functionality of BV-displayed scFvE2/p17 molecules**. Baculoviral progeny recovered from the culture medium of Sf9 cells infected with BV-E2/p17 was isolated by ultracentrifugation. **(a)**, BV-E2/p17 virions were immobilized on ELISA plate and reacted with anti-HA tag monoclonal antibody and peroxidase-labeled complementary antibody. Controls were from left to right, respectively: uncoated well, well coated with antigen with no BV addition, and well coated with antigen with addition of irrelevant baculovirus particles (CAR-displaying BV^CAR^; [41]). **(b)**, Schematic model of scFvE2/p17 molecule displayed at the surface of a BV particle. GP64, baculoviral envelope major glycoprotein. BV-E2/p17 virions were reacted in ELISA with immobilized antigen, **(c) **H_6_MA-CA-embedded p17 epitope, or **(d) **synthetic peptide p17.1. Average of three separate experiments, m ± SEM; (*), *P *< 0.05); (**), *P *< 0.01).

The observation that BV-displayed scFvE2/p17 was accessible to anti-HA tag antibodies in immuno-EM analysis strongly suggested that the C-terminal HA tag was oriented outwards. This orientation was already suggested by IF microscopy of intact cells expressing scFvE2/p17 (refer to Figure 2A, i). The next experiments were designed to assess the topological orientation of the scFvE2/p17 molecule in the baculoviral envelope, and, more importantly, to determine the degree of accessibility of its antigen-binding regions. BV-E2/p17 virions were immobilized on ELISA plate and probed with anti-HA tag antibody. The positive reaction indicated that the carboxyterminal region of scFvE2/p17 was exposed at the surface of the virions (Figure 7a), and that the insertion of scFvE2/p17 in the baculoviral envelope mimicked the orientation of class II membrane glycoproteins, with the aminoterminal region anchored in the baculoviral envelope, as depicted in Figure 7b.

The antigen binding activity of BV-displayed scFvE2/p17 was then determined by ELISA, using immobilized H_6_MA-CA protein or synthetic peptide p17.1. In this assay, BV-E2/p17 virions displaying scFvE2/p17 were used as the equivalent of primary antibodies, and bound virions were detected using monoclonal antibody directed towards the baculoviral glycoprotein GP64. Both H_6_MA-CA protein and p17.1 peptide were recognized by BV-E2/p17 virions (Figure 7c and 7d), which indicated that the BV-displayed scFvE2/p17 molecules retained their antigen-binding capacity.

### BV-display of HA-tagged anti-CD147 scFv-M6-1B9

**Figure 8:**
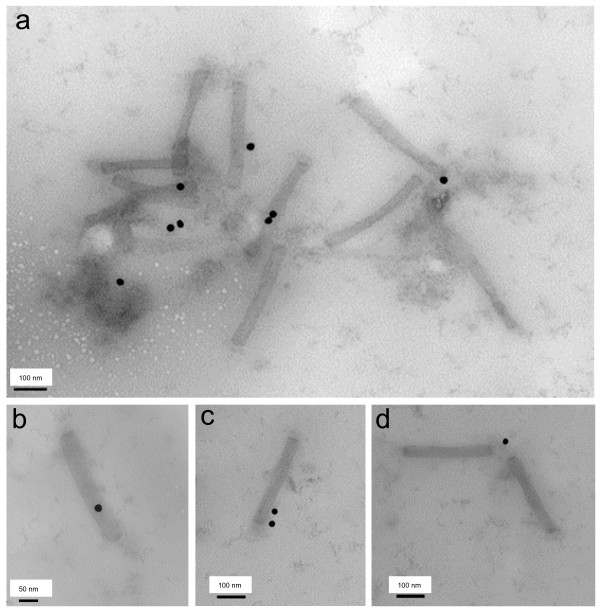
**Immuno-electron microscopy of BV particles carrying chimeric scFv-N18E2/M6 (BV-N18E2/M6)**. Samples of baculoviral progeny recovered at 72 h pi from the culture medium of Sf9 cells infected with BV-N18E2/M6 were deposited on grids and negatively stained with uranyl acetate. Specimens were reacted with anti-HA tag monoclonal antibody, followed by anti-mouse IgG antibody coupled to 20-nm colloidal gold grains. Different fields are presented in panels (a) to (d). **(a)**, General view of a cluster of immunogold-labeled BV-N18E2/M6 particles. **(b-d)**, Enlargement of immunogold-labeled BV-N18E2/M6 particles.

ScFv expressed in recombinant BV-infected insect cells are not naturally or spontaneously addressed to the baculoviral envelope, and different methods have been developed to obtain baculoviral envelope insertion of various scFv molecules, including fusion to GP64 or to VSV-G stem [16-21]. Although scFvE2/p17 did not contain any consensus signal peptide for membrane addressing, we postulated that in Sf9 cells, the N-terminal octadecapeptide sequence ^1^MEASLAAQAAQIQLVQSG^18 ^(abbreviated N18E2), was responsible for the intracellular trafficking and targeting of scFvE2/p17 to the site of BV budding, where it became inserted into the baculoviral envelope. To test this hypothesis, N18E2 was fused to the N-terminus of a non-related scFv, the HA-tagged scFv-M6-1B9, which recognizes the membrane glycoprotein CD147 [38,39]. When expressed in HeLa or 293 cells, scFv-M6-1B9 occurred as an active anti-CD147 intrabody, provoking the intracellular retention of CD147 and the blockage of its surface expression [38,39]. Our resulting chimeric scFv construct, abbreviated scFv-N18E2/M6, was expressed in Sf9 cells using a recombinant BV vector (BV-N18E2/M6). ScFv-N18E2/M6 molecules were detected in significant levels at the surface of nonpermeabilized cells harvested at 48 h pi, as for scFvE2/p17 (refer to Figure 2B). The baculoviral progeny from BV-N18E2/M6-infected cells was then isolated by ultracentrifugation and analyzed by ELISA (not shown) and immuno-EM (Figure 8) using anti-HA tag antibody. Both methods showed the accessibility of the HA tag at the surface of BV particles. In the electron micrograph shown in Figure 8d, two baculovirus particles were seen as a V-shape dimer pointing to one gold grain. This might represent the cross-linking of two particles via the two Fab domains of a single antibody molecule, although it could not be excluded that one antibody would bind to one particle, while the other would be in close proximity.

**Figure 9:**
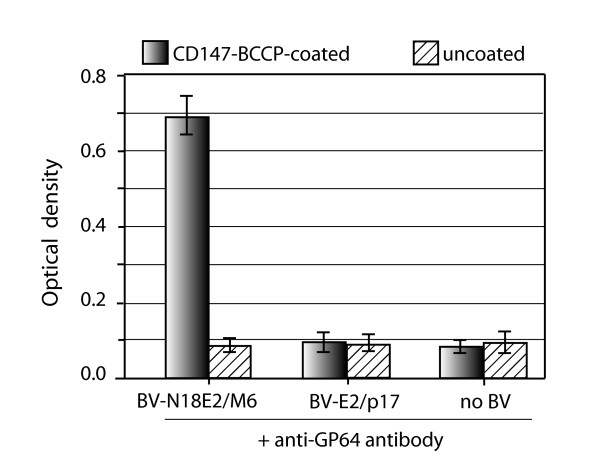
**Functional analysis of BV-displayed chimeric scFv-N18E2/M6**. The antigen-binding capacity and specificity of chimeric scFvE2/M6 displayed on the BV particle envelope was assessed by indirect ELISA, as described in a previous study [38,39]. Aliquots of BV-N18E2/M6 and control BV-E2/p17 particles recovered from BV-infected Sf9 cells were added to CD147-BCCP-linked avidin-coated wells, and incubated for 1 h at RT. After washing steps, bound viral particles were detected by addition of anti-baculoviral envelope glycoprotein GP64 in TBS-BSA. Average of three separate experiments, m ± SEM. (**), *P *< 0.01.

**Figure 10:**
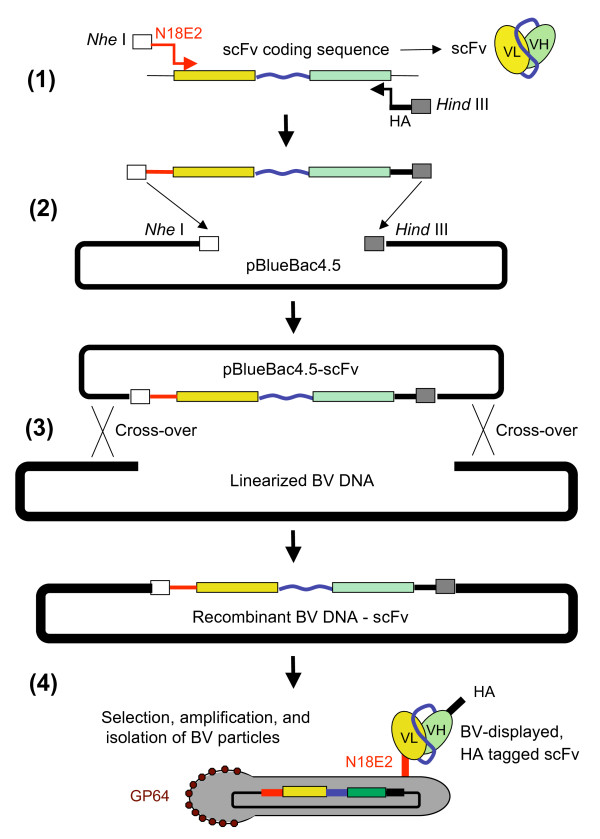
**Generation of recombinant BV vector designed for display of scFv using the N18E2 signal peptide**. Shown is the stepwise construction and isolation of BV-N18E2/scFv, a recombinant BV designed to expose at its surface scFv molecules of interest equiped with the signal octadecapeptide N18E2. ***Step (1) ***: PCR amplification of the scFv coding sequence, using a Fw 5'-primer containing a *Nhe *I site and the N18E2 coding sequence, and Rev 3'-primer encoding a HA tag and a *Hind *III site. ***Step (2) ***: insertion of N18E2-scFv-HA-encoding DNA fragment into the baculoviral intermediate plasmid pBlueBac4.5, digested with *Nhe *I and *Hind *III. ***Step (3) ***: cotransfection of insect cells with pBlueBac4.5-N18E2-scFv-HA and linearized baculoviral DNA, and homologous recombination. ***Step (4) ***: Isolation of plaques positive for recombinant baculoviral clone harboring N18E2-scFv-HA (e.g. blue plaque selection); amplification and isolation of recombinant BV expressing N18E2-scFv-HA, and displaying N18E2-scFv-HA on the baculoviral envelope.

We also tested the antigen-binding capability of BV-displayed chimeric scFv-N18E2/M6, using the recombinant CD147-BCCP fusion protein as immobilized antigen in ELISA, as previously described [39]. The data showed that the chimeric scFv-N18E2/M6 molecules present at the surface of BV particles conserved their antigen-binding function and their specificity towards CD147 (Figure 9). These results suggested that the fusion of the N18E2 peptide to scFv-M6-1B9 was able to confer to the chimeric scFv-N18E2/M6 protein the capacity to be addressed to the BV budding sites at the plasma membrane and to be incorporated into the baculoviral envelope. They also suggested that N18E2 could function as a universal N-terminal signal peptide for BV-display of functional scFv of biological interest. A scheme of the general strategy of stepwise construction of BV particles displaying scFv molecules of interest equiped with our signal octadecapeptide N18E2 is presented in Figure 10.

## Discussion

Despite several recent studies, little information is available at the molecular level on the molecular factors and mechanisms involved in the secretory pathway of insect cells in general, and the lepidopterian Sf9 cells in particular [46]. Secreted recombinant proteins are usually obtained by fusion of the protein of interest to the N-terminal leader peptide of the honeybee pro-mellitin (MBM-SP) [47,48]. However, this strategy does not always guarantee the secretion of the recombinant protein. For example, scSCR20, a MBM-SP-leaded scFv derived from an anti-African cassava mosaic virus monoclonal antibody [49] was not secreted in the culture medium of BV-infected insect cell lines from *Spodoptera frugiperda*, *Trichoplusia ni*, and *Mamestra brassicae*. However, scSCR20 molecules were released at high levels in the culture medum of *Drosophila *cell lines stably expressing MBM-SP-fused scSCR20 [50]. Likewise, certain types of scFv expressed in insect cells using recombinant BV have been recovered in the extracellular medium although they lack an insect cell leader peptide [51]. The most probable explanation was that specific features of recombinant scFv, such as their own N-terminal amino acid sequence or/and other downstream domain(s), can influence their behaviour in BV-infected cells.

BV-display has been used for almost a decade for immunisation purposes, gene delivery, or development of eukaryotic libraries [14,17,52,53]. Conventional BV-display involves baculoviral envelope glycoprotein GP64 manipulations [17,18,52], or the use of the VSV-G stem [16,17,20,21]. This differs from the incorporation of foreign proteins or glycoproteins into the baculoviral envelope without fusion to GP64, such as the envelope incorporation and display of functional human beta-2 adrenergic receptor (ß2AR) described in an earlier study [54]. In a more recent work, we demonstrated the incorporation of the human CAR glycoprotein into the baculoviral envelope. CAR is the high affinity receptor for adenovirus serotype 5 (Ad5) and is a resident glycoprotein of the human cell plasma membrane. The baculoviral envelope-incorporated CAR was fully functional at the surface of BV^CAR ^virions, and enabled the formation of BV^CAR^-Ad5 complexes, mediated by the interaction between the adenoviral fiber and CAR. We have used this strategy of BV^CAR^-Ad5 duo formation to transduce Ad5-refactory cells [41].

It was relatively easy to conceive that human ß2AR and CAR molecules, even though expressed in heterologous system, could be displayed on the baculoviral envelope since both are resident membrane glycoproteins. It was rather unexpected for scFvE2/p17, which was an artificial molecule extrinsic to the BV-insect cell system. Moreover, in the case of scFvE2/p17, the scFv molecule was not constructed for membrane targeting, in contrast to scFvG2/p17 which carried the specific Met-Gly dipeptide signal for N-myristoylation by N-myristoyl-transferases. Comparison of the amino acid sequences of scFvE2/p17 and scFvG2/p17, which both lacked any consensus leader peptide, showed that they only differed by three residues at their N-terminus, M(EAS)L for scFvE2/p17, versus M(G)L for scFvG2/p17. The results of these minor sequence changes were drastic in terms of scFv solubility, cell compartmentalization and extracellular release. Recombinant scFvG2/p17 protein expressed in Sf9 cells was inexploitable since it was insoluble and trapped in the membrane pellet.

Recombinant scFvE2/p17 however was recovered simultaneously under two different forms: (i) as soluble scFv molecules from lysates of BV-infected Sf9 cells, and (ii) as BV-displayed scFv in the culture medium of the BV-infected Sf9 cell cultures. Both forms could be used as biological tools for different purposes. Soluble scFvE2/p17 could serve in conventional diagnostic assays for HIV-1 Gag detection, through specific recognition of the conserved MAp17 epitope. MAp17 functions as a structural component of HIV-1 virions, but also as a viral cytokine which binds to a cellular receptor, p17R [55,56], when released by HIV-infected cells. In the case of BV-displayed scFvE2/p17, the potential applications would be different. For example, if one considers the virokine properties of soluble MAp17 and the importance of inflammatory response at the mucosal sites of HIV-1 entry [55,56], one could envisage to use pelletable, BV-displayed scFvE2/p17 in experimental models of infected mucosae to deplete soluble MAp17 from the extracellular medium, or/and to compete with MAp17 for binding to p17R.

To assess the role of the N-terminal domain of scFvE2/p17 in the process of membrane addressing and scFv display on the baculoviral envelope, we fused the N-terminal octadecapeptide ^1^MEASLAAQAAQIQLVQSG^18 ^(abbreviated N18E2) to another bioactive scFv molecule, scFv-M6-1B9. The ligand of scFv-M6-1B9 is M6, also called CD147 [38,39], a transmembrane glycoprotein highly expressed in various types of malignant cells [57] and tumors, e.g. nasopharyngeal carcinoma [58]. CD147 acts as an inducer of extracellular matrix metalloproteinases (EMMPRIN is another acronym for CD147) to promote tumor growth, invasion, metastasis and neoangiogenesis, and is a prognostic marker for invasiveness in prostate cancer [59] and thyroid carcinoma [60]. CD147 is also involved in atherosclerosis plaque instability [61] and in the regulatory inhibition of starvation-induced autophagy in human hepatoma cells [62].

We expressed the chimeric scFv-N18E2/M6 molecule in recombinant BV-infected Sf9 cells, and found that the BV progeny displayed scFv-N18E2/M6 on the baculoviral envelope. This suggested that the N-terminal octadecapeptide N18E2 carried the function required for BV-display of scFv molecules, and could be considered as a BV envelope addressing/anchoring signal peptide. This was further supported by the comparison of the structural domains of scFv downstream of the N18E2 peptide: scFv-N18E2/M6 and scFvE2/p17 differed by the successive order of their variable regions, VL-linker-VH from the N- to C-terminus in scFv-M6-1B9, versus VH-linker-VL in scFvE2/p17. This implied that the nature of the variable region downstream of N18E2 had little influence, if any, on the membrane addressing of chimeric, N18E2-fused scFv.

Although the molecular mechanism of cell trafficking of our chimeric scFv-N18E2/M6 molecule still remained to be elucidated in molecular terms, our present data provided a novel concept and platform for engineering scFv molecules competent for BV-display.

## Conclusion

In the present study, we identified a N-terminal octadecameric peptide sequence, N18E2, which mediated the plasma membrane addressing and anchoring of scFv into the baculoviral envelope, and acted as a BV-envelope display signal. N18E2 could therefore be used in a general technology for BV-display of bioactive molecules such as scFv. In previous studies, we provided evidence that scFv-M6-1B9 was biologically active as an intrabody, and could be used to diminish the expression of CD147 at the surface of human cells [38,39]. The data presented here showed that scFv-M6-1B9 was also functional when displayed on the BV vector envelope. One might therefore envisage to use scFv-N18E2/M6-displaying BV particles as CD147-targeted vectors in future protocols of cancer biotherapy, to transfer therapeutic genes (e.g. suicide genes or proapoptotic genes) to CD147-overexpressing malignant cells. Since the baculoviral envelope glycoprotein GP64 is a low specificity attachment protein which allows BV to enter a wide variety of cells originating from mammalian or nonmammalian species [14,41], it will be necessary to block the GP64 binding activity of such vectors, and/or place the desired therapeutic gene under the control of a tumor-specific promoter, in order to avoid any bystander effect on cells of the surrounding tissues.

## Authors' contributions

KK and SN carried out the genetic construction of recombinant baculoviral clones, the biological and functional characterization of the recombinant proteins, and participated in the discussions. GG carried out the flow cytometry and immunoelectron microscopy analyses. PB participated in the immunoelectron microscopy analyses and wrote the manuscript. SSH participated in the genetic constructs. CT, SSH and PB conceived the study, participated in its coordination and designed the experimental strategies. All authors read and approved the final manuscript.
